# Supplementary material for: The causal relationship between the human gut microbiota and pyogenic arthritis: a Mendelian randomization study
Source: Front Cell Infect Microbiol. 2024 Nov 26;14:1452480. doi: 10.3389/fcimb.2024.1452480 (PMC11629706; doi:10.3389/fcimb.2024.1452480)
Supplement: Supplementary file 1 [file DataSheet1.zip › mendelian test/ebi-a-GCST90027715.csv_leave_one_out.pdf]

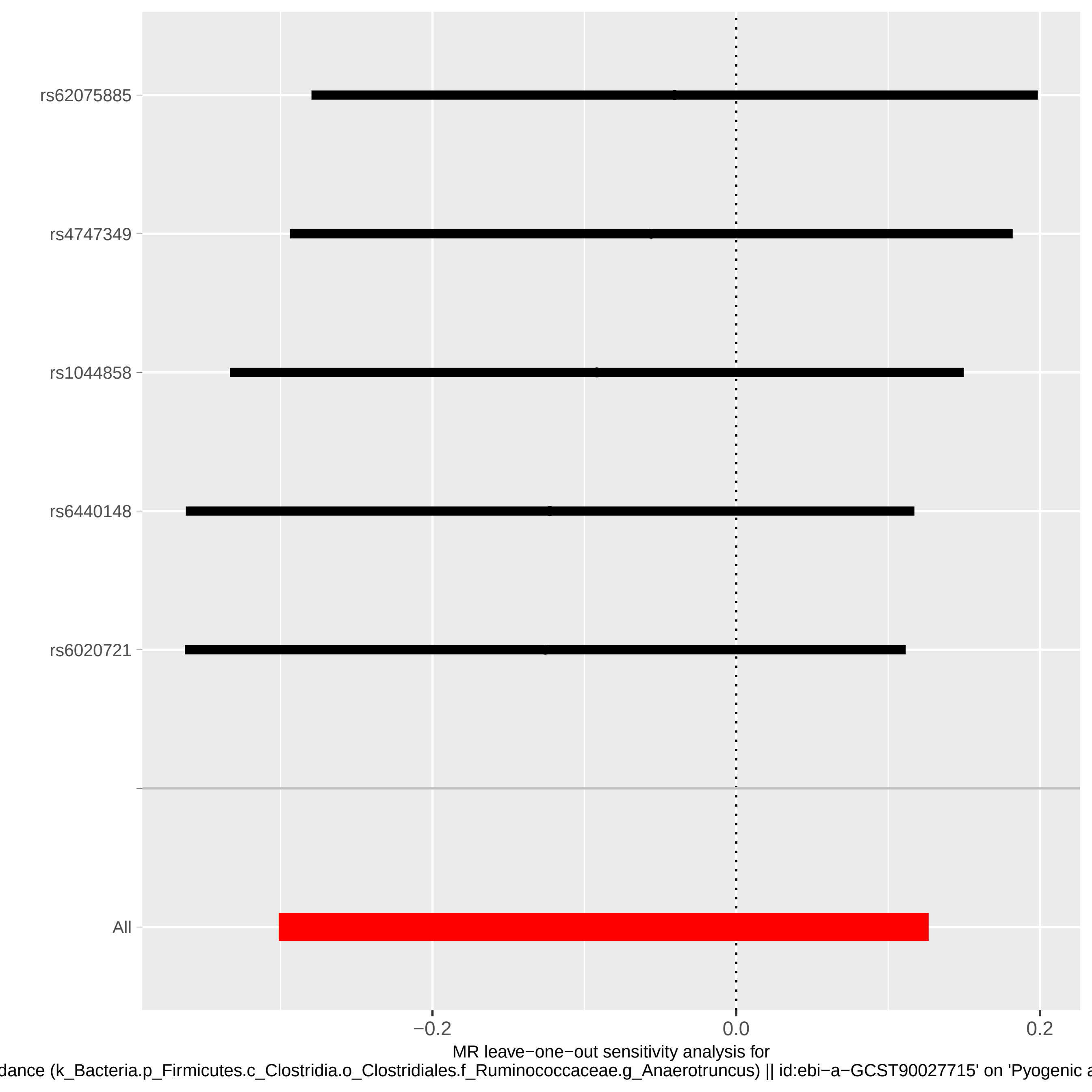
rs62075885
rs4747349
rs1044858
rs6440148
rs6020721
All
−0.2
0.0
0.2
MR leave−one−out sensitivity analysis for
dance (k_Bacteria.p_Firmicutes.c_Clostridia.o_Clostridiales.f_Ruminococcaceae.g_Anaerotruncus) || id:ebi−a−GCST90027715' on 'Pyogenic a
